# Health status among greenhouse workers exposed to different levels of pesticides: A genetic matching analysis

**DOI:** 10.1038/s41598-020-65662-1

**Published:** 2020-05-26

**Authors:** Yongxin Xie, Jiangping Li, Xin Guo, Ji Zhao, Biao Yang, Wenwen Xiao, Huifang Yang

**Affiliations:** 1 Department of Labor and Environmental Hygiene, School of Public Health and Management, Ningxia Medical University, Yinchuan, Ningxia Hui Autonomous Region 750004 China; 2 Department of Epidemiology and Statistics, School of Public Health and Management, Ningxia Medical University, Yinchuan, Ningxia Hui Autonomous Region 750004 China

**Keywords:** Diseases, Health care, Health occupations, Medical research, Risk factors

## Abstract

(1) Objective: Greenhouse workers are considered a special occupational group who are exposed to more toxic and harmful substances than ordinary farmers. The health problem of this group is a public health problem that warrants attention. Taking greenhouse workers in Ningxia, China, as the research sample, this study analyzed the health risk to practitioners posed by the greenhouse working environment. (2) Method: To analyze the relationship between pesticide exposure and the health of greenhouse workers, the genetic matching method was used to exclude the influence of covariates on the results. (3) Results: The results showed a statistical significance regarding the prevalence of cardiovascular diseases (CVD), skeletal muscle system diseases (SMSD) and digestive diseases between the different exposure groups. Researching the disease symptoms found that different levels of exposure to pesticides in greenhouses could cause multisystem and multisymptom discomfort. In addition to some irritant symptoms such as eye itching, itching, and sneezing, there were also differences in terms of the frequency of discomfort such as back pain, a decline in sleep quality, memory loss, joint pain, swelling and weakness, upper abdominal pain and flatulence, in the different exposure groups. (4) Conclusion: Different levels of exposure to pesticides in greenhouses may be one of the risk factors for practitioners to suffer from various systemic diseases, affecting their health and work efficiency. This hazard is manifested not only in some acute irritant symptoms but also in chronic diseases due to long-term exposure.

## Introduction

China is a large agricultural country. According to statistics, China’s rural population accounts for 40.42% of the total population^1^. Greenhouse are widely used in rural China as a planting method that is not affected by physical factors such as the season or the environment. Some studies have shown that greenhouse vegetable cultivation is becoming increasingly common in China, and the planting area is showing an increasing trend^2^. In Shouguang, China, alone, the annual output of greenhouse vegetables is 4.5 million tons^3^, and this output is an important sources of vegetables and fruits for many provinces and cities.

As greenhouse become a convenient way to provide resident with fresh vegetables, pesticide exposure is one of the risk factors for the health of greenhouse pratitioners that cannot be ignored. The emergence of the new large greenhouse (LSGH) has led to a significant increase in pesticide use intensity^4^. A study using WHO case definitions in Serbia has also suggested that greenhouse workers (56%) had a high incidence of acute pesticide poisoning within 12 months (30%)^5^. Another study pointed out that greenhouse operations were closely related to acute pesticide poisoning^6^. Long-term and high-intensity pesticide exposure, coupled with the high temperature and humidity in greenhouses, has caused different degrees of damage to the health of practitioners, involving various systems of the human body, such as the nervous^7–9^, reproductive^10^, respiratory^11^, circulatory^12^, digestive^13^, endocrine systems^14^ and so on. In response to those problems, the studies have reported that exposure to fenpropathrin can mimic the pathological and pathogenic characteristics of Parkinson’s disease (PD), suggesting that fenpropathrin is a dopamine neurotoxin and may be an environmental risk factor for PD^15^. Alessandra Antunes Dos Santos *et al*. believed that chronic exposure to malathion could lead to memory loss and a decline in spatial discrimination^8^. Analyzing human oral cells in Malaysia, Zariyantey found that compared with the control group (office workers), the frequency of manganese in farmers exposed to pesticides increased significantly^16^. Many studies have also shown that different levels of pesticide exposure have a certain cytotoxicity and genotoxicity^17,18^, which may lead to the occurrence of and deterioration due to multisystem diseases.

In a previous cohort study, we analyzed health problems related to multisystem diseases among greenhouse workers^19,20^. At the same time, besticide exposure in the greenhouse microenvironment was also analyzed and studied^21^. In this study, genetic matching methods are used. Potential confounding factors are well controlled as covariates to minimize the impact of bias on the results, better reflection the true, impact of pesticide exposure on the health of practitioners, and providing a theoretical basis for the health risk factors of greenhouse workers.

## Materials and Methods

### Data source

The data come from health survey data on greenhouse workers randomly sampled from four greenhouse planting villages (Wudu, Lingtian, Maosheng, Yinghe) in Yinchuan city in Ningxia, China in 2015, 2016 and 2017. The data were collected through face-to-face questionnaires with informed consent. The questionnaire is a self-compiled questionnaire from the research group, and after reliability and validity testing, the quality of the questionnaire was found to be good. The research plan was approved by the Medical Ethics Committee of Ningxia Medical University for the record (approval number 2014-090).

### Sample collection

According to the formula for sample size in simple random sampling,

$$N=\frac{{u}_{\alpha }^{2}(1-p)}{{\delta }^{2}}$$

where *p* is 30% and δ is controlled at 5%. The sample size needed for simple random sampling is 225. We expand the sample size 1.5 times based on cluster sampling and finally determine a sample size of not less than 403 every year based on a 20% attrition rate. The actual sample sizes in the three years are 448, 460, and 460 respectively.

The inclusion criteria are as follows:

1. The respondents are local residents (i.e., living in the local area for more than five year);
2. The respondents are part of the main labor force population aged 18–70;
3. The respondents have been engaged in the relevant work of greenhouse planting for no less than one year.

The exclusion criteria are as follows:

1. People who refused to participate in the investigation after communication;
2. People with cognitive impairment or who cannot communicate effectively;
3. People who can’t work continuously in Greenhouse.

### Matching

As a method of causal inference, matching is widely used in many fields, such as statistics, medicine, public health, economics, and sociology. Matching can control for potential confounding factors as covariates to minimize the impact of bias on the results. When using the matching method for causal inference, two common approaches are propensity score matching^22^ and multivariate matching based on the Mahalanobis distance^23,24^. Both methods need to have the attribute of “equal percent bias reduction” (EPBR)^25,26^, but this attribute is seldom consistent in real data. If it is not established, then the matching will increase the bias of some linear functions of the covariates even if all univariate means are closer in the matched data than in the unmatched data^27^. At the same time, these two methods also have the disadvantage of worsening the balance between potential confounding factors. Therefore, in 2005 and 2011, Sekhon and his colleagues proposed the genetic matching algorithm, which can maximize the balance of the observed covariates between the different exposure groups.

### Genetic Matching

Genetic matching is a nonparametric matching algorithm that was proposed by Sekhon and his colleagues^28,29^. Its core motivation for matching is also Rubin’s causal model^30^. It does not depend on the understanding and estimation of propensity scores; rather, it is a generalization of propensity score matching and Mahalanobis distance matching. The greatest advantage of genetic matching is that it can quickly find an appropriate weight by machine learning, so that the covariates involved in matching can reach the distribution balance between, the different exposure groups as soon as possible. Genetic matching does not need to establish a model to predict the tendency score value in advance; rather, genetic matching weights it based on the importance of variables, to improve the accuracy and efficiency of the matching. Genetic matching is based on generalizing the Mahalanobis metric and gives weight parameter w to optimize the matching process. The formula is:

1 $${\rm{GMD}}({{\rm{X}}}_{{\rm{i}}}{,{\rm{X}}}_{{\rm{j}}},{\rm{W}}){=\{({\rm{X}}}_{{\rm{i}}}-{{\rm{X}}}_{{\rm{j}}}{)}^{{\rm{T}}}{({{\rm{S}}}^{-\frac{1}{2}})}^{{\rm{T}}}{{\rm{WS}}}^{-\frac{1}{2}}{{({\rm{X}}}_{{\rm{i}}}-{{\rm{X}}}_{{\rm{j}}})\}}^{-\frac{1}{2}},$$

where W is a k × k positive definite weight matrix and ${{\rm{S}}}^{-\frac{1}{2}}$ is the Cholesky decomposition of S. The formula is ${\rm{S}}={({{\rm{S}}}^{-\frac{1}{2}})}^{{\rm{T}}}{{\rm{S}}}^{-\frac{1}{2}}$. Its weight assignment is 0 when the information of all covariates (confounding factors) is included in the propensity score or when the model can match better through the Mahalanobis distance.

### Variable selection

Grouping variables, i.e., the high-exposure group and the low-exposure group, were obtained by latent cluster analysis (LCA) perfomed by the members of the previous project team. In the latent cluster analysis, the latent variables we choose mainly include the following three parts:

1. Basic characteristics of greenhouse workers: the number of years working in a greenhouse, the per capita planting area, and the working time in a greenhouse/year.
2. Direct contact with pesticide spraying: the personal spraying of pesticides, the mixing of pesticides, the spraying mode, the spraying interval and the spraying duration of each pesticide.
3. Pesticide spraving protection and protection awareness: behavioral factors during pesticide spraying, personal protective equipment scores, personal hygiene, and inspection before and during pesticide spraying.

The matching variables include three aspects: general demographic characteristics (sex, age, ethnicity, cultural levels, etc.), habits and customs (smoking, drinking, exercise, etc.), and dietary habit (number of meals, category of meals, fruit, salt and etc.).

### Statistical analysis method

R 3.5.2 was used to analyze the data. The major package was the matching package that Sekhon and his colleagues developed. The significance level is defined as P < 0.05.

### Ethics declarations

During the investigation, the investigators carefully read the relevant contents of the Declaration of Helsinki and strictly abided by its contents. The privacy of the respondents should be conserved and informed consent should be given. This survey is in line with the relevant content of the Declaration of Helsinki. Combine with the characteristics and implementation of this study, the specific Helsinki principles are summarized as follows:

1. This study adheres to ethical standards, respects all groups, and protects their health and rights;
2. In this survey, the investigators have the duty to protect the lives and health of the subjects and to maintain their privacy and dignity;
3. Before the implementation of this investigation, we will submit the design and implementation of the field investigation to the ethics committee of Ningxia Medical University for examination, comment, guidance and approval;
4. The investigation was approved and filed by the Medical Ethics Committee of Ningxia Medical University (Approval No. 2014-090). (See the annex below for specific proof.);
5. The survey was conducted on the premise that the respondents could benefit from the results of the study;
6. All the respondents in this survey volunteered to participate in this survey, have a full understanding of the research project, and signed the informed consent from;
7. The survey promises to respect the rights of the respondents, to protect their privacy and to minimize any impact on their lives;
8. The purpose, method, source of funding, affiliated units of the researchers, expected benefits and potential risks of the survey were explained to each respondent;
9. This survey promises that the respondents can terminate the project at any time for any reason.

## Results

### General demographic characteristics of greenhouse workers

A total of 1368 individuals were selected for this study, including 448 in 2015, 460 in 2016 and 460 in 2017. After LCA, 392 people were included in the high-exposure group, and 976 were included in the low-exposure group, accounting for 28.65% and 71.35% of the total population, respectively. The working environment, pesticide use and personal protection of the greenhouse workers are described in Table 1.

**Table 1:** Descriptive analysis of working environment and pesticide use in greenhouse.

		Total	Percentage(%)
Greenhouse planting area	(Mean±SD)	4398.14	3.22 ± 4.02
Number of greenhouses planted (Mean±SD)		3057.65	2.24 ± 1.85
Working hours in the greenhouse every year	<50 days	5	0.37
	50–99 days	19	1.39
	100–199 days	221	16.15
	200–299 days	372	27.19
	>300 days	751	54.90
Mixed pesticide	No	273	19.96
	Occasionally	477	34.87
	Often	618	45.17
Spraying method of pesticides	Spray on insects	630	46.05
	Regular spraying	267	19.52
	Advance prevention	698	51.02
	Follow others	5	0.37
Spraying position of pesticides	According to the instructions	694	50.73
	Spray on the leaves until wet	114	8.33
	Drop pesticide on leaves	179	13.08
	Essays will be	77	5.63
	Depending on the condition	365	26.68
Spraying mode	Machine spraying	1227	89.69
	Artificial spraying	117	8.56
	Mixed spraying	24	1.75
Personal protective measures	No protective measures	470	34.36
	Wear protective mask	608	44.44
	Wear protective clothing	415	30.34
	Wear protective glasses	41	3.00
	Wear protective gloves	441	32.24
	Wear protective rubber shoes	94	6.87
Types of pesticides	Insecticide	1032	75.44
	Bactericide	1029	75.22
	Herbicide	196	14.33
	Foliar fertilizer	748	54.68
	Plant growth regulators	295	21.56
Whether to change clothes after spraying pesticide	Change clothesimmediately	826	60.38
	Change clothes on the day when you go home	410	29.97
	Uncertain	74	5.41
	No change clothes	58	4.24
Whether to take a bath after spraying pesticide	Immediately	518	37.87
	On the day when you go home	659	48.17
	Don’t take a bath on the day	191	13.96
Whether to wash hands after spraying pesticide	Immediately	1066	77.92
	On the day when you go home	244	17.84
	Don’t wash hands on the day	58	4.24

### Genetic matching

**Figure 1:**
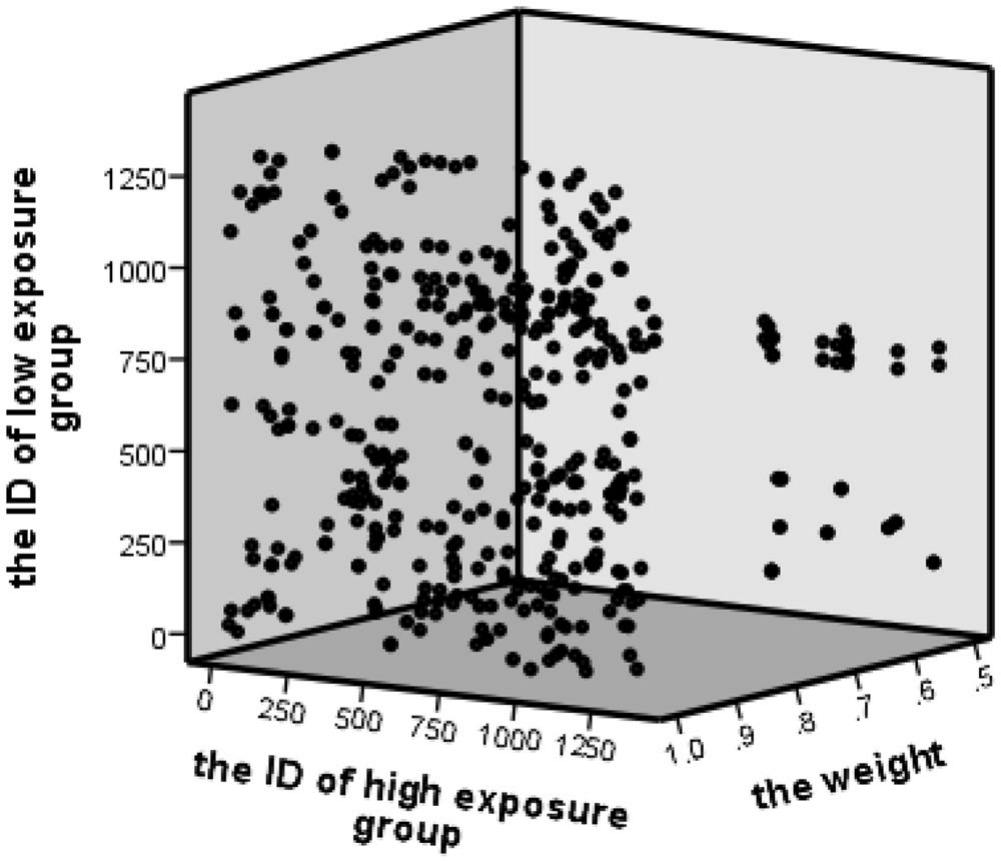
Basic information of genetic matching.

In this study, 392 pairs were successfully matched, with each pair having a certain weight. Based on the weight, the matched data were processed and analyzed. The bubble diagram in Fig. 1 shows the basic information after genetic matching of the sample data. The x-axis is the ID of the high-exposure group, the y-axis is the ID of the low-exposure group, and the z-axis is the weight. Notably, in the matching process, there are some high-exposure objects matching multiple low-exposure objects.

Table 2 shows the equilibrium test results before and after covariate matching. When all covariates were included in the matching, there was no significant difference between the high-exposure group and the low-exposure group. This result indicates that genetic matching eliminates the bias of covariates (confounding factors) in this data analysis to a certain extent.

**Table 2:** Equilibrium test results before and after covariate matching.

		Before matching		After matching	
		S.E of D-value^1^	P	S.E of D-value^1^	P
Family size		12.222	0.047	−2.409	0.221
Gender		5.026	0.400	1.529	0.467
Ethnic		−2.215	0.712	0.795	0.564
Age		0.870	0.884	3.521	0.340
Education		−15.272	0.011	−4.244	0.222
Marital status		−0.462	0.938	−0.752	0.317
Length of residence		5.737	0.344	6.046	0.199
Income group		3.670	0.540	−0.451	0.907
**Living habits**					
	Smoking	5.488	0.359	5.761	0.174
	Second-hand smoke exposure	−7.886	0.192	−0.614	0.904
	Drinking	−3.206	0.592	−2.738	0.679
	Exercise	3.532	0.551	7.535	0.227
**Dietary habits**					
	Number of meals per day	−12.000	0.043	−1.863	0.285
	Eating breakfast	3.619	0.541	3.001	0.325
	Eating meat	−7.673	0.176	−2.575	0.157
	Eating fresh vegetables	4.146	0.515	2.924	0.317
	Eating fresh fruits	−17.973	0.001	0.000	1.000
	Eating pickles	−12.717	0.032	1.040	0.414
	Salt	−15.795	0.008	−1.910	0.303

1. S.E of D-value means standard error of difference value between high and low exposure groups.

### Differential analysis of diseases

Before matching, there was no significant difference in the prevalence of cardiovascular diseases (CVD) between the different exposure groups (p = 0.059), but after matching, the prevalence of CVD was different between the different exposure groups (p = 0.009). The results suggest that pesticide exposure in a vegetable greenhouse environment had an effect on the CVD after excluding interference by covariates. Furthermore the analysis show s that after matching, the prevalence of CVD in the high-exposure group (7.91%) was higher than that in the low-exposure group (3.57%), about which it can be concluded that the occupational environment of the vegetable greenhouse was one of the causes of CVD. In the analysis of skeletal muscle system diseases (SMSD), the same conclusion is drawn: before matching, there was no difference in prevalence between the different exposure groups (p = 0.059), but after matching, there was a significant difference in prevalence between the different exposure groups (p = 0.013). Meanwhile after matching, the prevalence rates of the high- and low-exposure groups were 15.56% and 9.69%, respectively. This result indicates that after eliminating the interference of other covariates, the working environment of the vegetable greenhouse is one of the causes of SMSD. Rdgardless of whether before or after matching, there were no significant differences in the prevalence of digestive, respiratory or immune-endocrine diseases between the different exposure groups. There was a statistically significant difference in the prevalence of digestive system diseases between the two groups before and after matching; however, for respiratory system diseases and immune-endocrine system diseases, there was no significant difference. The results are shown in Table 3.

**Table 3:** Difference test of diseases in major human systems before and after matching between different exposure Groups.

		Before matching				After matching			
		Low exposure	High exposure	*χ*^2^	*P*	Low exposure	High exposure	*χ*^2^	*P*
Cardiovascular system				3.572	0.059			6.813	0.009
	Yes	925	361			378	361		
	No	51	31			14	31		
Skeletal muscle system				3.572	0.059			6.116	0.013
	Yes	846	331			354	331		
	No	130	61			38	61		
Digestive system				8.079	0.004			7.354	0.007
	Yes	829	308			337	308		
	No	147	84			55	84		
Respiratory system				0.199	0.656			1.39	0.238
	Yes	927	370			377	370		
	No	49	22			15	22		
Immune and endocrine system				0.157	0.692			0.065	0.799
	Yes	895	362			361	362		
	No	81	30			32	30		

### Difference analysis of symptoms

Comparing the difference in the frequency of stimulus symptoms, we found that there was no significant difference between the different exposure groups before and after matching. Notably, however, there were significant differences in symptoms of eye discomfort (itching, pain, dry eyes, etc.) and unexplained sneezing and runny nose between the different exposure groups. However, there was no significant difference between asthma and skin discomfort. The specific results are shown in Table 4.

**Table 4:** Difference Test of Frequency of Stimulation Symptoms before and after Matching.

		Before matching				After matching			
		Low exposure	High exposure	χ2	P	Low exposure	High exposure	χ2	P
Red swelling and fever of the skin				3.155	0.368			3.201	0.362
	Nothing	809	315			329	315		
	Light	128	54			46	54		
	Middle	38	23			16	23		
	High	1	0			1	0		
Eye pruritus				15.102	0.001			8.038	0.018
	Nothing	777	279			313	279		
	Light	140	68			49	68		
	Middle	59	45			30	45		
Unexplained sneezing and runny nose				7.141	0.028			8.165	0.017
	Nothing	732	274			294	274		
	Light	205	90			87	90		
	Middle	39	28			11	28		
Asthma				3.01	0.222			2.18	0.336
	Nothing	921	360			370	360		
	Light	40	23			17	23		
	Middle	15	9			5	9		

Comparing the differences in cardiovascular symptoms, we found that before matching, the symptom of right back pain did not differ in the different exposure groups (p = 0.065), however, after matching, excluding the confounding of other covariates, there was a significant difference between the different exposure groups (p = 0.047). Then the frequency of the high-exposure group was higher than that of the low-exposure group. The results are shown in Table 5.

**Table 5:** Difference Test of Symptom Frequency in Cardiovascular System before and after Matching.

		Before matching				After matching			
		Low exposure	High exposure	*χ*^2^	*P*	Low exposure	High exposure	*χ*^2^	*P*
Chest pain				0.276	0.871			1.573	0.455
	Nothing	855	345			335	345		
	Light	104	39			50	39		
	Middle	17	8			7	8		
Right back pain				5.469	0.065			6.099	0.047
	Nothing	840	318			342	318		
	Light	110	58			42	58		
	Middle	26	16			8	16		
Dyspnea				1.877	0.391			0.517	0.772
	Nothing	903	355			356	355		
	Light	66	32			33	32		
	Middle	7	5			3	5		
Nausea and vomiting				3.637	0.162			3.375	0.185
	Nothing	915	358			371	358		
	Light	49	30			18	30		
	Middle	12	4			3	4		
Edema of lower limbs				0.574	0.751			1.349	0.509
	Nothing	937	373			378	373		
	Light	26	12			7	12		
	Middle	13	7			7	7		
Cyanosis				5.167	0.075			2.2	0.333
	Nothing	957	381			381	381		
	Light	19	9			11	9		
	Middle	0	2			0	2		
Anxiety and irritability				2.762	0.251			1.163	0.559
	Nothing	869	344			350	344		
	Light	101	42			39	42		
	Middle	6	6			3	6		

Comparing the differences in nervous system symptoms, we found that before matching, there was no significant difference in the distribution of sleep quality between the different exposure groups (p = 0.228), but after matching, the difference was statistically significant. The frequency of poor sleep quality in the high-exposure group was significantly wider than that in the low-exposure group. The distribution of sleep time and memory impairment showed the opposite results. Before matching, the distribution of sleep time in the high-exposure group was significantly wider than that in the low-exposure group. At the same time, the symptoms of difficulty falling asleep, nightmares and sleep pain that were not affected by covariates were statistically significant in the different exposure groups before and after matching. Table 6 suggests that after matching, the frequency of three symptoms, i.e., difficulty falling asleep, nightmares and sleep pain, was significantly higher in the high-exposure group than in the low-exposure group.

**Table 6:** Difference Test of Symptom Frequency in nervous system before and after Matching.

		Before matching				After matching			
		Low exposure	High exposure	*χ*^2^	*P*	Low exposure	High exposure	*χ*^2^	*P*
Sleep time	Mean±sd	7.64 ± 1.42	7.82 ± 1.39	−2.148	0.032	7.72 ± 1.41	7.82 ± 1.39		0.310
Sleep quality				4.328	0.228			14.208	0.003
	Good	525	202			234	202		
	Fine	309	136			108	136		
	Poor	117	50			36	50		
	Very poor	25	4			15	4		
Drug hypnosis				0.570	0.903			3.001	0.391
	Nothing	966	387			386	387		
	<1 times/week	6	3			2	3		
	1–2 times/week	3	1			4	1		
	>3 times/week	1	1			0	1		
Difficulty in falling asleep				7.938	0.047			12.580	0.006
	Nothing	757	278			320	278		
	<1 times/week	111	60			41	60		
	1–2 times/week	43	26			13	26		
	>3 times/week	65	28			19	28		
Difficulties breathin				4.808	0.186			4.667	0.198
	Nothing	914	354			369	354		
	<1 times/week	36	21			13	21		
	1–2 times/week	18	11			8	11		
	>3 times/week	8	6			2	6		
Nightmare				8.058	0.045			9.653	0.022
	Nothing	668	237			276	237		
	<1 times/week	149	77			56	77		
	1–2 times/week	79	38			24	38		
	>3 times/week	80	40			36	40		
Sleep pain				21.630	0.001			22.866	0.001
	Nothing	842	305			354	305		
	<1 times/week	72	55			24	55		
	1–2 times/week	37	12			8	12		
	>3 times/week	25	20			7	20		
Lack of physical strength				2.670	0.445			1.714	0.634
	Nothing	522	192			209	192		
	Light	348	154			137	154		
	Middle	105	45			45	45		
	High	1	1			1	1		
Visual impairment				2.563	0.278			2.471	0.291
	Nothing	618	230			252	230		
	Light	237	107			94	107		
	Middle	121	55			47	55		
Hypomnesia				10.964	0.012			3.251	0.355
	Nothing	570	197			219	197		
	Light	269	120			108	120		
	Middle	137	74			66	74		
	High	0	1			0	1		
Loss of interest in things				0.164	0.921			1.015	0.602
	Nothing	674	270			281	270		
	Light	250	99			87	99		
	Middle	52	23			24	23		
Headache and vertigo				2.982	0.225			0.397	0.820
	Nothing	642	240			245	240		
	Light	257	121			114	121		
	Middle	77	31			34	31		

Comparing the difference of skeletal muscle system symptoms, we found that before matching, there was no significant difference in the distribution of pain, swelling and weakness symptoms between the different exposure groups, however, after matching, the incidence of symptoms in the high-exposure group was higher than that in the low-exposure group (p = 0.024). The results are shown in Table 7.

**Table 7:** Difference Test of Symptom Frequency in Skeletal muscle system before and after Matching.

		Before matching				After matching			
		Low exposure	High exposure	*χ*^2^	*P*	Low exposure	High exposure	*χ*^2^	*P*
Pain and swelling of fingers or toes of unknown origin				1.376	0.502			2.089	0.352
	Nothing	774	314			316	314		
	Light	157	56			62	56		
	Middle	44	22			14	22		
Pain, swelling and weakness in the joints of hands and feet				3.062	0.216			7.472	0.024
	Nothing	713	267			296	268		
	Light	180	86			77	86		
	Middle	83	38			20	38		
Muscle soreness and pain in the whole body				0.121	0.941			1.411	0.494
	Nothing	669	266			281	266		
	Light	218	88			79	88		
	Middle	89	38			32	38		

In the difference analysis of digestive system symptoms, only after matching were the symptoms of upper abdominal pain and flatulencesignificantly different between the different exposure groups (p = 0.038). The results are shown in Table 8.

**Table 8:** Difference Test of Symptom Frequency in Digestive system before and after Matching.

		Before matching				After matching			
		Low exposure	High exposure	*χ*^2^	*P*	Low exposure	High exposure	*χ*^2^	*P*
Upper abdominal pain and flatulence				1.043	0.594			6.560	0.038
	Nothing	865	352			350	352		
	Light	95	32			41	32		
	Middle	16	8			1	8		
Nausea, vomiting and acid regurgitation				2.472	0.291			3.823	0.148
	Nothing	881	358			355	358		
	Light	85	27			35	27		
	Middle	10	7			2	7		
Abnormal stool				1.343	0.511			1.201	0.549
	Nothing	914	361			366	361		
	Light	47	22			21	22		
	Middle	15	9			5	9		

In the difference comparison of respiratory symptoms, only after matching was the incidence of unexplained hemoptysis significantly different in the different exposure groups (p = 0.001). The results are shown in Table 9.

**Table 9:** Difference Test of Symptom Frequency in Respiratory system before and after Matching.

		Before matching				After matching			
		Low exposure	High exposure	*χ*^2^	*P*	Low exposure	High exposure	*χ*^2^	*P*
Cough and expectoration				2.251	0.325			0.968	0.616
	Nothing	830	323			334	323		
	Light	127	57			49	57		
	Middle	19	12			10	12		
Shortness of breath				2.993	0.224			1.442	0.486
	Nothing	906	353			363	353		
	Light	62	34			27	34		
	Middle	8	5			3	5		
Chest tightness and shortness of breath				1.936	0.38			0.755	0.685
	Nothing	889	347			354	347		
	Light	74	38			33	38		
	Middle	14	7			5	7		
Unexplained hemoptysis				—	0.054			10.473	0.001
	Nothing	960	391			379	391		
	Middle	16	10			13	1		

There was no significant difference in other variables, including the immune and endocrine systems before and after matching. The results are shown in Table 10.

**Table 10:** Difference Test of Symptom Frequency in Immune and endocrine system before and after Matching.

		Before matching				After matching			
		Low exposure	High exposure	*χ*^2^	*P*	Low exposure	High exposure	*χ*^2^	*P*
Cold				2.178	0.336			0.546	0.761
	Nothing	634	242			244	242		
	Light	295	134			129	134		
	Middle	47	16			20	16		
Blood does not clot easily				0.269	0.874			0.049	0.976
	Nothing	941	380			379	380		
	Light	30	10			11	10		
	Middle	50	2			2	2		

## Disscusion

As a special working environment, vegetable greenhouses are characterized by a closed environment, pesticides and other toxic substances do not easily volatilize. Therefore, there Studying the health problems of vegetable greenhouse practitioners holds a certain level of scientific and practical value^31,32^. Health is the result of a combination of factors related to genetics, the environment, living habits and other factors^33^. If a single-factor method is used to study the principle of health occurrence and development, such a method will have certain limitations, and it will not be possible to exclude the interference of mixed factors in real causality. In this study, genetic matching was used to eliminate the covariate bias between disease and environmental exposure in greenhouses, and to explore the relationship between different diseases (symptoms) and environmental exposure in greenhouses.

Genetic matching is a new matching method subsequent to propensity score matching. It can quickly find an appropriate weight by machine learning so that the covariates involved in matching can reach the distribution balance among groups as soon as possible^30^. Its matching speed and quality are so fast that those of previous matching methods cannot compare. In this study, the equilibrium test of genetic matching also pointed out that there was no significant difference in all covariates between the high- and low-exposure groups after matching, indicating that the matching effect was good.

After genetic matching, it was statistically significant for the difference of the CVD among the different exposure groups, and the prevalence of the CVD in the high-exposure group was higher than that in the low-exposure group, showing that the degree of exposure to greenhouse pesticides had a causal relationship with the CVD of workers after excluding the interference of covariate factors. The Global Burden of Disease (GBD) study indicated that the CVD have been a major cause of global mortality since 1980^34^. In 2015, CVD accounted for nearly one-third of all deaths worldwide, while such diseases accounted for more than 40% of all deaths in China^35,36^. Previous studies by our group have also suggested that the CVD, as one of the high-risk diseases of vegetable greenhouse practitioners, are still a problem that cannot be ignored^30^. Comparing the symptoms of CVD in the different exposure groups, we found that after matching, the occurrence frequency of right back pain was different between the different exposure groups and always showed that the occurrence frequency of the high-exposure group was higher than that of the low-exposure group. There were no differences between the different exposure groups regarding other symptoms. This result may be because CVD have an acute onset, there are more temporary discomfort symptoms in the early stage^37^, and the body itself has a certain tolerance, coupled with differences in individual cognition. Therefore it is easy to ignore the degree of concern for such symptoms.

People engaged in agricultural labor usually maintain a certain forced position in the process of labor, which in the long run will cause skeletal muscles fatigue and induce disease. Studying SMSD among greenhouse workers, it was also found that there were differences in the prevalence of SMSD among greenhouse workers with different exposure levels, and the prevalence of such diseases in the high-exposure group was always higher than that in the low-exposure group. This result is consistent with Zhang’s research^33^. With the increase in exposure intensity, the working intensity of workers is also increasing, making some of the skeletal muscles of workers be in a long-term state of tension, and the risk of disease will continue to increase. Some studies have also shown that the prevalence of osteoarthritis in greenhouse workers was 41.9%^38^, which was much higher than that of other workers. It can be seen that SMSD are one of the important factors affecting the health status of greenhouse workers. In addition, the study of disease-related symptoms indicated that the prevalence of the symptoms, “pain, swelling and weakness of hand and foot joints”, were significantly higher in the high-exposure group than in the low-exposure group, which also indicated that joints such as those in the hands and feet of greenhouse workers were important body parts prone to injury, and were body parts that need to be emphatically protected^39^.

Many pesticides can harm the digestive system^40^. In this research, the prevalence of digestive system diseases in the high-exposure group was higher than that in the low-exposure group before and after matching. After matching, the prevalence of such diseases was 21.43% and 15.06% in the high- and low-exposure groups respectively, which was higher than other researchers’ survey results on digestive system diseases in rural residents^41^. This result further indicates that the vegetable greenhouse environment has a promoting effect on digestive system diseases compared with the ordinary rural working environment. In the digestive symptom analysis, upper abdominal pain and flatulence were different between the different exposure groups, and the frequency of the high-exposure group was higher than that of the low-exposure group. On the one hand, this result may be due to improper protective measures and other factors. Pesticides are more likely to enter the body through the mouth and nose when greenhouse workers spray pesticides, and pesticides entering the body will bind to serine in the center of pancreatic cholinesterase activity. This binding will inhibit acetylcholine activity and thus result in a large amount of accumulation of acetylcholine in nerve synapses, affecting the nerve conduction function, and causing the gastrointestinal function to fail to function well^42^. On the other hand, greenhouse workers need to carry a certain volume of pesticide spraying cans when spraying pesticides, which will cause their abdomen to be in a state of long-term oppression, and therefore, these workers will be more likely than the general population to exhibit abdominal distension, abdominal pain and other discomfort symptoms. Some scholars have also suggested that^43^ pesticide exposure was associated with the occurrence of digestive system diseases. As a result of the effects and high frequency of pesticide spraying and exposure to the special microenvironment of greenhouses, pesticide exposure in greenhouses is one of the risk factors for digestive system diseases.

Studying neurological symptoms, we found that with the increase in the frequency of pesticide exposure in greenhouses, workers will suffer from different degrees of neurological discomfort, such as a decline in sleep quality, difficulty falling asleep, sleep pain, and memory loss. Research has shown that the nervous system is another important target organ for pesticide exposure. People exposed to pesticides may have symptoms of neurological discomfort of varying degrees, and the symptoms may be aggravated with the increase in exposure intensity^44,45^. Because of the special working environment, greenhouses aggravate damage to the nervous system of practitioners and make them more prone to symptoms of discomfort. Therefore, as one of the risk factors for digestive system diseases, greenhouse pesticide exposure should be given sufficient attention by researchers. In addition, different levels of exposure to pesticides can cause different degrees of eye itching, sneezing and other irritant symptoms^46,47^. Effective protection is an effective way to alleviate pesticide irritant symptoms in greenhouses. Reducing the frequency and intensity of exposure can effectively alleviate the irritant symptoms^47^.

In this research, it was shown that there was no difference in symptoms related to the respiratory system and the endocrine system between the different exposure groups, which is inconsistent with some researchers’ findings^14,48^. This result may be due to the short period of time working in a greenhouse for the workers in the selected sample areas; additionally, the sample population is not engaged in this kind of work all year round. They enter greenhouses to do related work only when greenhouses are places busy with farming activity. And other times, they choose to go out for other work without exposure to pesticides. This period serves as the elution period of pesticide exposure toxicity. Therefore, the impact of the greenhouse environment on the disease incidence of different systems will be weakened, but follow-up research should be conducted to verify this hypothesis.

## Conclusion

It was found that exposure to different degrees of greenhouse pesticides can not only lead to multisystem diseases in human, but also cause many uncomfortable physical symptoms for greenhouse practitioners, affecting their health and work efficiency. This hazard was manifested not only in some acute irritant symptoms, but also in chronic diseases due to long-term exposure^18^.

## References

[1] Statistics, N. B. O. Statistical Yearbook of Chin, China Statistics Press (Beijing, 2019).

[2] Ping M (2016). Problems to be Grasped in Optimum Design of Vegetable Greenhouse. Structure. Chinese Melon and vegetable..

[3] Yun, Z. Why Shouguang Became a Vegetable Town? (2008) Available at, http://www.ctoutiao.com/953427.html (Accessed: 11.

[4] Beyene, N., Hans, K., Yalemtshay, M. & Roel, V. Use of Chemical Pesticides in Ethiopia: A Cross-Sectional Comparative Study on Knowledge, Attitude and Practice of Farmers and Farm Workers in Three Farming Systems. *The Annals of occupational hygiene*. **60** (2016). 10.1093/annhyg/mew004 26847604

[5] Nigatu, A. W., Bråtveit, M. & Moen, B. E. Self-Reported Acute Pesticide Intoxications in Ethiopia. *BMC Public Health*. **16** (2016). 10.1186/s12889-016-3196-5 PMC4946227 27422555

[6] Samuel, F. *et al*. Exposure to Pesticides and Health Effects on Farm Owners and Workers From Conventional and Organic Agricultural Farms in Costa Rica: Protocol for a Cross-Sectional Study. *JMIR research protocols*. **8** (2019). 10.2196/10914 PMC6367668 30681969

[7] Motsoeneng PM, Dalvie MA (2016). Relationship Between Urinary Pesticide Residue Levels and Neurotoxic Symptoms Among Women On Farms in the Western Cape, South Africa. Int. J. Env. Res. Pub. He..

[8] Antunes, D. S. A. *et al*. Long-Term and Low-Dose Malathion Exposure Causes Cognitive Impairment in Adult Mice: Evidence of Hippocampal Mitochondrial Dysfunction, Astrogliosis and Apoptotic Events. *Arch. Toxicol*. **90** (2016). 10.1007/s00204-015-1466-0 25618550

[9] Shala, C. *et al*. A Prospective Cohort Study of School-Going Children Investigating Reproductive and Neurobehavioral Health Effects Due to Environmental Pesticide Exposure in the Western Cape, South Africa: Study Protocol. *BMC Public Health*. **18** (2018). 10.1186/s12889-018-5783-0 PMC6042376 29996806

[10] van Wendel De Joode, B. *et al*. Pesticide Exposure and Neurodevelopment in Children Aged 6–9 Years From Talamanca, Costa Rica. *Cortex*. **85** (2016). 10.1016/j.cortex.2016.09.003 27773359

[11] Kingsley IA, Sunday IP, Blessing AA (2015). Histological assessment of the Effects of PyrethroidsinsecticideMorteinon the Lungs of Adult WistarRats. IOSR Journal of Dental and Medical Sciences..

[12] Silva JF, Mattos IE, Luz LL, Carmo CN, Aydos RD (2016). Exposure to Pesticides and Prostate Cancer: Systematic Review of the Literature. Reviews on Environmental Health..

[13] Xiangmin L (2010). Analysis On Correlated Clinical Factors of Upper Gastrointestinal Tract Bleeding Induced by Acute Organophosphorus Pesticide Poisoning. Chinese. Journal of Practical Internal Medicine..

[14] C-J, Y. *et al*. Increased Risk of Attention-Deficit/Hyperactivity Disorder Associated with Exposure to Organophosphate Pesticide in Taiwanese Children. *Andrology-US*. **4** (2016). 10.1111/andr.12183 27070915

[15] Jing, X. *et al*. Fenpropathrin, a Widely Used Pesticide, Causes Dopaminergic Degeneration. *Mol. Neurobiol*. **53** (2016). 10.1007/s12035-014-9057-2 PMC5333774 25575680

[16] Zariyantey, A. H. *et al*. The Association of Nuclear Abnormalities in Exfoliated Buccal Epithelial Cells with the Health Status of Different Agricultural Activities Farmers in Peninsular Malaysia. *Genes and environment: the official journal of the Japanese Environmental Mutagen Society*. **38** (2016). 10.1186/s41021-016-0032-1 PMC4918015 27350827

[17] Dutta S, Bahadur M (2016). Cytogenetic Analysis of Micronuclei and Cell Death Parameters in Epithelial Cells of Pesticide Exposed Tea Garden Workers. Toxicol. Mech. Method..

[18] Hans-Peter, H. *et al*. Cytotoxic and Genotoxic Effects of Pesticide Exposure in Male Coffee Farmworkers of the Jarabacoa Region, Dominican Republic. *Int*. *J. Env. Res. Pub. He*. **15** (2018). 10.3390/ijerph15081641 PMC6121533 30081446

[19] Xiaoyu Z, Bing W, Lijun D, Ji Z, Huifang Y (2017). Cross-Sectional Survey On Genitourinary System Health of Vegetable Greenhouse Growers in Suburban Areas in Yinchuan. Journal of Occupational and Environmental Health..

[20] Lijun D (2017). Analysis On Present Status and Risk Factors of Digestive System Diseases in Greenhouse Growers On Outskirts of Yinchuan City. Chinese Journal of Industrial Medicine..

[21] Jian S (2014). Current Status of Organophosphorus Pesticide Residuesin Vegetables Grown in Greenhouse in Yinchuan Suburb. Journal of Environmental Hygiene..

[22] Rosenbaum PR, Rubin DB (1983). The Central Role of the Propensity Score in Observational Studies for Causal Effffects. Biometrika..

[23] Rubin DB (1980). Bias Reduction Using Mahalanobis-Metric Matching. Biometrics..

[24] Cochran, W. G. & Rubin, D. B. Controlling Bias in Observational Studies: A Review William G. Cochran and Donald B. Rubin. *The Indian Journal of Statistics*. 417-446 (1973).

[25] Rubin DB (1974). Multivariate Matching Methods that are Equal Percent Bias Reducing, II: Maximums On Bias Reduction for Fixed Sample Sizes. ETS Research Bulletin Series..

[26] Rubin DB, Thomas N (1992). Affinely Invariant Matching Methods with Ellipsoidal Distributions. Ann. Stat..

[27] Rubin DB (1976). Multivariate Matching Methods that are Equal Percent Bias Reducing, I: Some Examples. Biometrics..

[28] W. R. M., Sekhon JS (2011). Genetic Optimization Using Derivatives: The Rgenoud Package for R. J. Stat. Softw..

[29] Diamond A, Sekhon HU (2012). Genetic Matching for Estimating Causal Effects: A General Multivariate Matching Method for Achieving Balance in Observational Studies. Review of Economics & Statistics..

[30] Sekhon JS (2011). Multivariate and Propensity Score Matching Software with Automated Balance Optimization: The Matching package for R. J. Stat. Softw..

[31] Yang L (2014). The Impact of Greenhouse Vegetable Farming Duration and Soil Types On Phytoavailability of Heavy Metals and their Health Risk in Eastern China. Chemosphere..

[32] Hu, W., Chen, Y., Huang, B. & Niedermann, S. Health Risk Assessment of Heavy Metals in Soils and Vegetables from a Typical Greenhouse Vegetable Production System in China. *Human and Ecological Risk Assessment: An International Journal*. **20** (2014).

[33] Zhang, M., Wang, X. F., Cui, X. M., Wang, J. & Shi-Xin, Y. U. Health Status of Solar Greenhouse Workers and Indoor Ambient in Vegetable Production Areas of Shandong Province. *Journal of Environmental & Occupational Medicine*. (2014).

[34] Institute For Health Metrics IHME. Institute for Health Metrics and Evaluation (IHME) (2017) GBD Compare Data Visualization. (2017) Available at, http://ghdx.healthdata.org/record/ihme-data/gbd-2017-cause-specific-mortality-1980-2017 (Accessed: 03 spetember 39).

[35] Barber, R. M. *et al*. Healthcare Access and Quality Index Based On Mortality From Causes Amenable to Personal Health Care in 195 Countries and Territories, 1990–2015: A Novel Analysis From the Global Burden of Disease Study 2015. *The Lancet*. **390** (2017). 10.1016/S0140-6736(17)30818-8 PMC5528124 28528753

[36] ≪Chinese Cardiovascular Disease Report 2017≫ Abstract. *Journal of Circulation in China*. (2018).

[37] Sameh, S., Anders, J. & Martin, S. What is Killing? People’s Knowledge About Coronary Heart Disease, Attitude Towards Prevention and Main Risk Reduction Barriers in Ismailia, Egypt (Descriptive Cross-Sectional Study). *The Pan African medical journal*. **15** (2013). 10.11604/pamj.2013.15.137.1628 PMC3853340 24319527

[38] Wenjing Z (2017). Prevalence of Musculoskeletal Diseases in Knee and its Influencing Factors Among Greenhouse Farmers in Shouguang. Chinese Journal of Disease Control..

[39] Qingfeng Z, Yuanyuan L, Jie X, Yugang Q (2010). Survey On Health Status of Vegetable Farmers in Greenhouse. Journal of Environment and Health..

[40] Namık, K., Mustafa, I. M., Bİöülent, S. & Sİöüükrİöüüüü, B. Influence of Pesticide Exposure On Carbonic Anhydrase II From Sheep Stomach. *Toxicol. Ind. Health*. **31** (2015). 10.1177/0748233713475508 23377119

[41] Muravyev, K. A. [the Rate and Dynamics of Prevalence of Diseases of Digestive System in North Caucasus Federal Okrug and Stavropolsky Kray]. (2011). 22611979

[42] Chunfang, S., Yunxiu, H., Xiying, L., Zhihuai, L. & Fenghua, F. Tth Effect of Plant Oil in the Absortion of Organophosphorous Pestieide in G Astrointestinal. *Chinese Journal of Modern Medicine*. 34–36 (2003).

[43] Kovtiuh L, Pavone E (2000). Chronic Pesticide Exposure and Increased Risk of Gastrointestinal Diseases. Epidemiology..

[44] Motsoeneng, P. M. & Dalvie, M. A. Relationship Between Urinary Pesticide Residue Levels and Neurotoxic Symptoms Among Women On Farms in the Western Cape, South Africa. *Int*. *J. Env. Res. Pub. He*. **12** (2015). 10.3390/ijerph120606281 PMC4483701 26042367

[45] Colosio, C., Mandic-Rajcevic, S., Rubino, F. & Brambilla, G. Emerging Health Effects From Pesticide Exposure in Europe and in Developing Countries. *Toxicol. Lett*. **205** (2011).

[46] Ergonen, A. T., Salacin, S. & Ozdemir, M. H. Pesticide Use Among Greenhouse Workers in Turkey. *Journal of Clinical Forensic Medicine*. **12** (2004). 10.1016/j.jcfm.2004.10.017 16054007

[47] Esechie, J. O. & Ibitayo, O. O. Pesticide Use and Related Health Problems Among Greenhouse Workers in Batinah Coastal Region of Oman. *J. Forensic Leg. Med*. **18** (2011). 10.1016/j.jflm.2011.02.009 21663866

[48] Walaa, D. *et al*. Impact of Chronic Exposure to the Pesticide Chlorpyrifos On Respiratory Parameters and Sleep Apnea in Juvenile and Adult Rats. *Plos one*. **13** (2018). 10.1371/journal.pone.0191237 PMC5777649 29357379

